# Induction and Transcriptional Regulation of Laccases in Fungi

**DOI:** 10.2174/138920211795564331

**Published:** 2011-04

**Authors:** Alessandra Piscitelli, Paola Giardina, Vincenzo Lettera, Cinzia Pezzella, Giovanni Sannia, Vincenza Faraco

**Affiliations:** University of Naples “Federico II”, Dipartimento di Chimica Organica e Biochimica, Complesso Universitario Monte S. Angelo, via Cintia 4, 80126 Napoli, Italy

**Keywords:** ACE1, aromatic compounds, copper, MRE, nitrogen, promoter, XRE.

## Abstract

Fungal laccases are phenol oxidases widely studied for their use in several industrial applications, including pulp bleaching in paper industry, dye decolourisation, detoxification of environmental pollutants and revalorization of wastes and wastewaters. The main difficulty in using these enzymes at industrial scale ensues from their production costs. Elucidation of the components and the mechanisms involved in regulation of laccase gene expression is crucial for increasing the productivity of native laccases in fungi. Laccase gene transcription is regulated by metal ions, various aromatic compounds related to lignin or lignin derivatives, nitrogen and carbon sources. In this manuscript, most of the published results on fungal laccase induction, as well as analyses of both the sequences and putative functions of laccase gene promoters are reviewed. Analyses of promoter sequences allow defining a correlation between the observed regulatory effects on laccase gene transcription and the presence of specific responsive elements, and postulating, in some cases, a mechanism for their functioning. Only few reports have investigated the molecular mechanisms underlying laccase regulation by different stimuli. The reported analyses suggest the existence of a complex picture of laccase regulation phenomena acting through a variety of cis acting elements. However, the general mechanisms for laccase transcriptional regulation are far from being unravelled yet.

## 1. LACCASE INDUCTION

Fungal laccases are phenol oxidases belonging to the group of multi-copper enzymes that catalyze the oxidation of a great variety of phenolic compounds and aromatic amines using molecular oxygen as electron acceptor [1]. Almost all the fungi examined secrete more than one laccase isozyme [1]. Due to their action over a broad range of substrates, fungal laccases have been widely studied for their use in several industrial applications, including pulp bleaching in paper industry, dye decolourisation, detoxification of environmental pollutants and revalorization of wastes and wastewaters [2-4]. Research on regulation of laccase gene expression may be very useful for increasing the productivity of native laccases in fungi and also for unravelling the physiological role of the different isoforms produced by the same organism. Synthesis and secretion of laccases are strictly influenced by nutrient levels, culture conditions, developmental stage as well as the addition of a wide range of inducers to cultural media, with variations among both different fungal species and different isoforms in a same strain. The effect of these factors at the level of laccase gene transcription has been demonstrated in many fungal species. Laccase gene transcription is often regulated by metal ions [5, 6], various aromatic compounds related to lignin or lignin derivatives [7], nutrient nitrogen [5] and carbon [8]. In most of the reported examples, laccase expression is regulated by an array of factors, acting in a synergistic or antagonistic way [9-12]. Physiological mechanisms occurring during mycelia development can also modulate the relative expression levels of laccase isoenzymes. Some isoforms have been observed during the lag and exponential phases of fungal fermentation and should be involved in substrate degradation, whilst other isoforms have been detected in the stationary phase and should be related to mushroom morphogenesis and pigmentation processes [13, 14].

### 1.1. Laccase Induction by Metals 

Regulation of laccase expression by metals is widespread in fungi. In *Trametes versicolor* 290, the highest laccase activity value (2500 U L^-1^) was achieved in cultures grown in the presence of 400 μM Cu^2+^, corresponding to a 18-fold higher level than in the absence of copper [5]. Galhaup and Haltrich [15] have studied the stimulatory effects of copper on the laccase production by different *Trametes* *spp*. The laccase activity of *Trametes versicolor* MB 52, *Trametes versicolor* MB 54, *Trametes suaveolens* MB 51 was induced by 1 mM Cu^2+^, reaching values of about 9000, 10000, and 7000 U L^-1^, respectively, while none of these strains produced laccase activity when grown on culture medium without additional Cu^+2 ^[15]. Lorenzo *et al*. [16] studied the effect of copper on laccase production of *Trametes versicolor* (CBS100.29). The highest values were obtained by cultures supplemented with 3.5 mM Cu^2+^, showing an increase of 11-fold and maximum values around 8000 U L^-1^. More recently, a higher laccase production induced by copper was observed by the *Trametes sp*. 48424. In this case, the highest laccase activity in the presence of 1 mM Cu^2+^ was 15273 U L^-1^ [17].

Even in *Pleurotus spp.* the induction of laccase by copper has been widely documented. Giardina *et al*. [18] obtained a laccase production of 30000 U L^-1^, growing *Pleurotus ostreatus* (ATCC MYA-2306) in nutrient-rich medium, with addition of 150 µM CuSO_4_, whereas laccase production resulted between 0.5 and 4 U L^-1^ in the presence of copper traces. More recently, a laccase production of 70000 U L^-1 ^was observed when this fungus was grown in semi-synthetic growth media formulated with agro-industrial by-products and supplemented with 150 µM CuSO_4_ (unpublished data). 

In all the analysed fungi (*T. versicolor* [5], *Ceriporiopsis subvermispora* [19], *P. ostreatus* [20, 11], *P. sajor-caju* [8], *Coriolopsis rigida* [3] and *Trametes pubescens* [6]), regulation of laccase production by copper has been demonstrated to occur at transcription level. In *P. ostreatus *(ATCC MYA-2306), Cu^2+^ has a marked effect on the induction of the examined isoenzymes POXC, POXA3 and POXA1b. The effect of Cu^2+^on laccase synthesis can be explained not only at transcriptional level. The results of Palmieri *et al*. [21] showed that Cu^2+^ concentration of 1 mM decreases the extracellular proteolytic activity, thus impairing laccase degradation.

Ag^+^, and Mn^+2^ ions have also been reported as modulators of laccase transcription [8,10]. It is worth noting that the same metal can exert opposite effects in different fungal species [10]. As a matter of fact, Mn^+2^ acts as an inducer of laccase transcription in *P. sajor caju* [8], *Clitocybula dusenii* and *Nematoloma frowardii *[22], but it was shown to inhibit laccase expression in *C. subvermispora* [10]. 

### 1.2. Laccase Induction by Aromatic Compounds

Phenolic and aromatic compounds structurally related to lignin or lignin derivatives are routinely added to fungal cultures to increase laccase production [23, 24]. Laccase induction by phenolic substances may represent a response developed by fungi against toxic aromatic compounds. By catalyzing their polymerization, laccases play a defensive role, reducing the oxidative stress caused by oxygen radicals originating from the reaction of these molecules [25]. An overview of the inductive responses by several fungi to different aromatic compounds is reported in Table **1** [26-47]. Laccase induction varies with the organism and seems to be specific to certain aromatic compounds.

The effect of veratryl alcohol on laccase production varies also with the composition of basal media used [48], enhancing laccase production from 2- to 200-fold. Guaiacol supplementation (1 mM) enhances laccase production from 2- to 232-fold in different fungi, giving maximum stimulatory effect in *Phlebia spp.* followed by *P. ostreatus* [24]. However, there is also evidence of suppression of laccase production by guaiacol in *Pycnoporus cinnabarinus* [41]. 

Among the 15 different phenolic compounds tested in *P. pulmonarius*, ferulic acid and vanillin produce the highest levels of laccase activity, inducing the expression of *lcc3* isoenzyme, not detectable in basal condition [23]. Screening of exogenous aromatic compounds for their effect on *P. ostreatus *laccase induction has shown ABTS (0.5-1 mM) as the most significant inducer, enhancing laccase activity production up to ﬁve-fold. 2,5-Xylidine, guaiacol and ferulic acid (1 mM) also increase laccase activity from two to three-fold, whereas veratryl alcohol does not stimulate enzyme production [24]. Moreover, the presence of ferulic acid (2 mM) in liquid culture of *P. ostreatus* (ATCC MYA-2306) also produces a different laccase isoenzyme pattern [20]. 

Laccase induction has also been demonstrated in the presence of dyes, the induction level being highly sensitive to small differences in their chemical structures [49]. 

Besides molecular structures and concentration, the inductive effect of phenolic compounds depends also on the time of their addition to the cultures [50]. In *P. pulmonarius* and *Botryosphaeria rhodina* cultures, the induction seems to be more efficient when the inducer was added to the cultures at inoculum time [23, 51], whereas in *P. ostreatus,* *Rigidoporus lignosus* and *Trametes modesta* medium conditioning is usually performed after 2-5 days from inoculation in order to avoid growth inhibition [52, 53]. High concentrations (>1 mM) of exogenous aromatic compounds in culture medium can negatively affect the fungal growth if the mycelium physiological state is not yet adapted to the changes of growth conditions or if it is compromised by environmental or mechanical stresses (preinoculum homogenization or sub-culturing from agar plate to liquid culture).

The induction of aromatic compounds has been found to occur at transcriptional level, differing not only among the various fungi analyzed, but also among the different isonzymes of the same organism. In *T. versicolor*, both 1-hydroxybenzotriazole and 2,5-xylidine effectively activate *lcc* transcription, whereas no induction is observed in the presence of either veratric acid or ferulic acid [5]. Transcription of one laccase gene, *lcc1*, from *T. villosa* is induced by the addition of 2,5-xylidine, but a second gene is constitutively expressed under the tested conditions [54]. In the ligninolytic basidiomycete *Trametes* sp. I62, nine structural closely related aromatic compounds appear to have different effects on laccase gene expression. The three laccase genes of this fungus are differentially expressed in response to these compounds, with specific induction patterns being observed for each molecule tested [7]. 

In the basidiomycete *Trametes* sp. AH28-2, where three novel laccase genes, *lacA*, *lacB*, and *lacC*, have been recently identified, it has been reported that different aromatic compounds can selectively induce the production of distinct laccase isozymes, with o-toluidine inducing the expression of LacA and 3,5-dihydroxytoluene mainly stimulating the production of LacB. Analysis by competitive Real Time Polymerase Chain Reaction showed that the accumulation of laccase RNA transcripts is accompanied by the increase of corresponding enzyme activity in cultures [55]. 

In *Volvariella volvacea*, *lcc1* transcript titres are differentially tuned by the addition of various aromatic compounds, whereas *lcc4* transcription is not affected by these molecules [56]. 

### 1.3. Laccase Regulation by Nitrogen and Carbon Sources

Laccase activity has also been shown to be dependent on the concentration and nature of carbon and nitrogen sources as well as on their ratio. Nitrogen source plays a key role in laccase production, with effects depending on its nature and concentration in culture media [41]. Change in laccase activity in response to nitrogen concentration is a controversial issue, since examples of activity increases have been described under both limiting and non-limiting conditions. Generally, inorganic nitrogen sources lead to low levels of laccase with sufficient biomass production, while organic nitrogen sources give high laccase yields with good fungal growth. Yeast extract is one of the best nitrogen sources that increases the yield of laccase enzymes [31]. The enzyme yield is also increased by supplementation of the medium with an additional nitrogen source like the amino acid L-asparagine [57].

Laccase induction at transcriptional level has been reported to occur under non-limiting nitrogen levels in the ligninolytic basidiomycete I62 (CECT 20197), where *lcc1* and *lcc2* transcript levels increased 100-fold in comparison with limiting nitrogen conditions [58]. Expression of *lcc1* in *Tramtes trogii* is preferentially induced by organic nitrogen sources compared with inorganic ones [59]. Individual laccase genes in *P. sajor-caju* are differentially induced at the transcriptional level by nitrogen sources, with a positive regulation by nitrogen occurring for *lac2* and *lac4*, and no effect being detected for *lac1 *and *lac3* [8]. 

As far as carbon sources are concerned, it has been demonstrated that supplementation of substrates, like glucose that are readily utilizable and efficiently metabolized by the microorganism, results in high levels of laccase activity [15]. Studies of laccase optimization in *P. ostreatus* IMI 395545 submerged cultures [12] have shown that glucose leads to the highest production of laccase compared to other carbon sources. As a matter of fact, fivefold increase of the laccase activity has been reported to occur under glucose concentration up to 20 g L^-1^.

In some fungi, laccase expression has been found to be subjected to catabolite repression: this mechanism is thought to be an energy-saving response. High carbon levels (around 15 g L^-1^) inhibit laccase transcription in basidiomycete I62 [58], *T. pubescens* [6], and *Trametes sp.* AH28-2 [55]. 

## 2. LACCASE PROMOTERS SEQUENCE ANALYSIS

*	In silico *inspection of several laccase promoter sequences highlights the presence of many different responsive elements differentially distributed along the promoter sequences. Promoter analyses allowed a correlation between the observed regulatory effects on laccase gene transcription and the presence of specific responsive elements, and to postulate in some cases, a mechanism for their functioning. Differences in copy number, location or orientation of the putative response elements determine a complex picture of laccase regulation phenomena.

The promoter region of the gene coding for the major laccase isoenzyme LAP2 from *T. pubescens*, extending about 1420 bp upstream of the start codon ATG, has been analyzed by Galhaup *et al. *(2002) [6]. This upstream region contains the usual promoter elements i.e. TATA box and seven CAAT motifs, along with two MREs (Metal Responsive Element), four CreA consensus sequences, 27 potential HSEs (Heat shock elements) [60] and a “general” STRE (Stress responsive element). Expression of LAP2 was found to be highly induced by copper and other heavy metal ions but repressed by glucose. Metal effect can be related to the presence of multiple MREs, causing a high level of induction [61]. *Cis*-acting MREs have been discovered in multiple copies in the *Saccharomyces cerevisiae* metallothionein promoter where they are essential for efficient metal-inducible transcription [62]. The presence of putative CreA-binding sites in the *lap2* promoter region suggests the existence of a carbon catabolite repressor similar to the CreA isolated in *A. nidulans* [63]. HSE and STRE might be involved in stress-regulated *lap2* gene expression caused, for example, by high concentrations of copper. Interestingly, neither xenobiotic response elements (XREs) nor antioxidant response elements (ARE) could be detected in the *lap* upstream region in accordance with the lack of transcriptional induction observed in the presence of aromatic substances [6].

Four putative MRE consensus sequences have been also found in the promoter region (~ 1kb long) of the copper inducible LAC2 laccase from *Gaeumannomyces graminis*, two of which are inverted and 51bp apart. In addition, this promoter contains two putative ACE1 (activation of cup1 expression protein) binding sites situated between the TATA box and ATG codon [64]. First described in *S. cerevisiae*, ACE binding site is the recognition site for the ACE1 copper-responsive transcription factor, which binds its target sequence in a copper-loaded conformation, activating the transcription of several genes [65]. An ACE binding sequence has also been found in the promoter of the copper induced laccase *lcs* from *C. subvermispora* [19].

Soden and Dobson [8] have performed an in-depth study on the promoter regions of four laccase genes (*lac1, lac2, lac3 *and* lac4*, extending 724bp, 214bp, 840bp and 1740bp upstream of the start codons, respectively) from *P. sajorcaju* displaying differential regulation in response to different stimuli [66]. A number of putative response elements including MRE, XRE, ARE (Antioxidant response element) and HSE have shown to be differentially distributed in the analysed promoter regions. The presence of *consensus* sequences putatively involved in nitrogen and carbon regulation such as CreA, Mig and NIT2 [67] binding sites, may explain the regulation by nutrient carbon and nitrogen sources, detected for some *P. sajor-caju* laccase genes [8], mediated by Mig1p or NIT2- like proteins. A single MRE has been identified in the promoter region of the strongly copper-induced *lac4* gene, whilst sequences with perfect homology with XRE have been detected in *lac1* and *lac4* promoters. Interestingly, laccase transcriptional analysis indicated that both *lac1* and *lac4* are regulated by the addition of 2,5-xylidine and ferulic acid, while *lac2* and *lac3*, both of which appear to lack XRE, are constitutively expressed under these conditions.

The differential expression of three laccase genes (*lacA*, *lacB* and *lacC*) from *Trametes sp. *AH28-2, has been investigated [55]. An inferred TATA box and several putative CAAT, MRE, XRE and CreA *consensus* sequences have been identified in the *lacA*, *lacB* and *lacC* promoter regions (1881bp, 993bp and 1703bp long, respectively). Differences in copy number and distribution of XREs among the three genes (seven XREs in *lacA* and only two in *lacB* and *lacC*) are supposed to be responsible for their different responses to aromatic compounds: *lacA* is induced by all tested aromatic compounds, *lacB* is induced mainly by guaiacol and 3,5-dihydroxytoluene, whilst *lacC* transcript levels are not detectable in the presence of these compounds. The overlapping of one XRE element with the TATA box in the *lacC* promoter is probably responsible for the absence of induction of this gene by aromatic compounds.

In addition to the above reported basidiomycetes, the presence of putative MREs has also been discovered in laccase promoter genes from the aquatic fungus *Myrioconium* sp. UHH 1-13-18 [68], and in the ascomycete *Hortea acidophila* [69] implying their potential role in copper induction of laccase expression.

The 1718 bp 5’-upstream region of a new laccase gene from *Trametes* sp. 48424 has been inspected by Fan and colleagues and compared with that of other known laccase genes from *Trametes* species [17]. This analysis highlighted the presence of new putative regulatory sites in the *lac48424-1* promoter region. Two putative iron responsive elements and three putative cAMP-responsive elements have been mapped in the upstream region, suggesting a role for cAMP in transcriptional regulation of laccase genes, as already proved for lignin and manganese dependent peroxidases in *Phanerochaete chrysosporium* [70]. Finally, the potential contribution of the Skn7 response regulator, a factor involved in the oxidative stress response in *S. cerevisiae* [71], in controlling laccase expression, has been hypothesized by the presence of two putative Skn7 regulatory elements in the *lac48424-1* promoter region. 

**Fig. (1):**
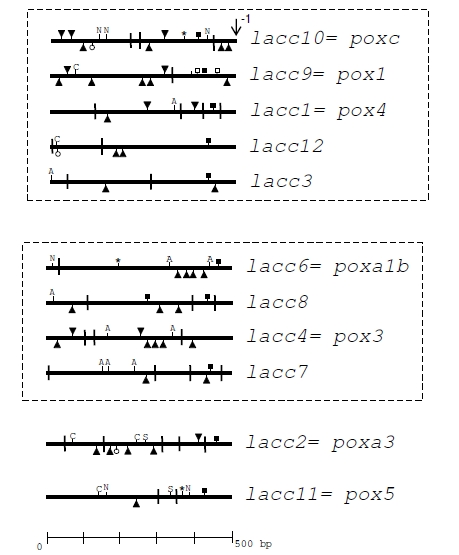
Distribution of putative cis-acting elements in the promoter regions of *P. ostreatus* laccase genes 500bp upstream of the start codons (↓): (■) TATA box; (|) CAAT box; (▼) GC box; (*) HSE (repeated NGAAN motif) [60]; (N) NIT2 (TATCT) [67]; (A) ARE (TGACNNNGC) [66]; (□) Putative response elements PRE (ATATC and TGGGT motifs) [66]; (▲) MRE; (O) XRE (TNGCGTG) [55]; (C) Cre-A-binding site (GCGGGG) [64]; (S) STRE Stress responsive element (CCCCT) [6].

Twelve putative laccase genes have been identified in the recently sequenced *P. ostreatus* genome (http://www.jgi.doe.gov/sequencing/why/50009.html), one of which is annotated as a ferroxidase-like. The sequence similarity and the intron–exon structure, along with the degree of identity among the corresponding protein sequences, allowed us to individuate two “laccase subfamilies”, while *poxa3* gene is evolutionary distant from both subfamilies [72]. The promoter regions of all *P. ostreatus* laccase genes, extending 500bp upstream of the start codon, have been analysed, revealing very little identity degree among them. *In silico* analysis allowed the identification of several putative response elements, differentially distributed along the promoter sequences (Fig. **1**). It is worth noting that the promoters of the most abundantly produced proteins (POXC and POXA3) display the highest number of signals belonging to different classes. Furthermore some characteristics are peculiar to each subfamily, i.e. the high number of ARE, and the absence of XRE and C and N nutrient responsive element in the *poxa1b* sub-family. 

## 3. FUNCTION OF LACCASE PROMOTERS

Despite the extensive data available by *in silico* analysis of many laccase promoters, only a few reports go through the molecular mechanisms underlying laccase regulation by different stimuli.

Some reports have been devoted to the comprehension of molecular aspects leading to laccase induction by copper. In *P. ostreatus *(ATCC MYA-2306), the formation of protein complexes on specific MREs identified in the promoter regions of the two copper induced laccase genes *poxc* and *poxa1b *[20], was verified by electromobility shift assays (EMSA) and foot-printing analysis [11]. EMSAs allowed the evaluation of the ability of the identified MREs to bind proteins from fungal extracts: all putative MREs in the *poxc* and *poxa1b* promoters (four MREs in each promoter region) are recognized by fungal proteins, except for one MRE in *poxc* promoter region, that is located downstream of the transcription-initiation site. Interestingly, the formation of complexes between the identified MREs and fungal proteins was found to occur in the absence of metal ions, suggesting the involvement of a 25 kDa negative-acting regulatory factor able to repress gene expression when bound to laccase promoter. Moreover, the role of specific nucleotides of the identified MREs in complex formation has been analysed, leading to the determination of an affinity scale of the tested MREs and to the definition of the optimal binding sequence. Results of UV-cross-linking analyses indicated that the highest affinity sequence binds also a 30 kDa factor. However, when a GC-rich region is located adjacent to the MRE site, this interaction is inhibited by the binding of a 15 kDa protein. A similar behaviour was also found in some metallothionein promoters where a regulatory role of Sp1, binding to this GC-rich region, in MRE-mediated transcriptional activation has been demonstrated [73].

An ACE1-like factor has been isolated in *P. chrysosporium* (Pc-ACE1-like) trough complementation of a *S. cerevisiae* *ace1∆* strain [74]. This factor was found to bind, in the presence of cuprous ions, its target ACE sequence in the promoter region of *mco1* gene, inducing gene expression. Using an *in vitro* transcriptional activation system, it was also demonstrated that Pc-ACE1-like is able to activate the transcription of a promoter-reporter construct in the presence of only one ACE element [75]. Intriguingly, copper supplementation to *P. chrysosporium* cultures also caused an increase of *mco2* mRNA levels, although *mco2* promoter regions lacks ACE elements. This effect may be ascribed to a different mechanism, possibly involving the generation of reactive oxygen species [75].

An ACE1-like factor was also isolated from *C. subvermispora *(Cs-ACE1-like). Specific binding of this factor to its cognate ACE sequence in the promoter of the copper induced *lcs* laccase was demonstrated by EMSA assays. This factor exhibited structural differences along with a lower complementation efficiency of the above mentioned *S. cerevisiae* strain, in comparison with its ortologue from *P. chrysosporium *[19]. In particular, the Cs-ACE1-like factor is considerable smaller than Pc-ACE1, lacking the second half of the CRD (Copper regulatory domain) present in ACE1 from *S. cerevisiae* and its ortologues. Although binding capacities of Cs-ACE1 to ACE elements is unaltered, its structural features may be responsible of the rather weak complementation of the *S. cerevisiae* *ace1 ∆* strain.

Mechanisms driving copper regulation have been also analysed in the pathogenic fungus *C. neoformans*, where LAC1 is a copper regulated laccase acting as a virulence factor [76]. In this fungus, copper induction of laccase expression was found to require the copper-responsive factor-encoding gene CUF1, since its disruption abolishes metal effect. The cryptococcal Cuf1 shares peptide sequence homology and similar biological functions to the counterparts from a few other fungi [77], harbouring the canonical copper-fist DNA binding region within its N-terminus that is common to the factors belonging to the ACE1 family. Laccase expression in *C. neoformans *was also found to be induced during glucose starvation, stimulated by copper, iron and calcium and repressed at elevated temperatures [78]. The complexity of laccase regulation is suggested by the presence of an extensive 1.3 kb 5’upstream region within the LAC1 gene, which contains three interacting enhancing sites, HSE and MRE sites and a novel GC-rich element (GCrE) immediately upstream to the HSE. The GCrE binding protein was purified and identified to be a Hsp70 homologue, Ssa1. Deletion of Ssa1 resulted in reduced laccase expression and attenuated virulence using a mouse model, indicating that Ssa1 functions as a stress-related transcriptional co-activator required for fungal virulence. Ssa1 was found to form a regulatory complex with heat shock transcription factor (HSF) and TATA-binding protein during laccase induction. As Ssa1 contains no activation domain of its own, a possible role for Ssa1 in laccase transcriptional activation is that its chaperone activity leads to conformational changes of HSF (that is able to constitutively bind to the HSE element in the laccase promoter region), allowing the formation of an active regulatory complex inducing laccase expression [79]. 

In plant pathogens, ambient pH may affect the expression of putative virulence factors, including laccases. Responses to pH variations occur *via *a conserved signalling cascade whose terminal component is the zinc-finger transcription factor PacC/Rimp1p [80]. PacC is a key component acting both as a transcriptional activator of alkaline-expressed genes and repressing acid-induced genes. The construction of two *pacC* mutants mimicking acidity or alkaline phenotypes allowed to infer the involvement of PacC in modulating laccase transcription in the plant pathogenic fungus *F. oxysporum*. As a fact, expression of *lcc3* occurs in acidic conditions and is down regulated by PacC, while expression of *lcc5* occurs only at pH 6 and is up regulated by the same factor. The presence of PacC binding consensus sites in *lcc3* and *lcc5* promoter regions further supports these data.

Oxidative stress response has been investigated in the fungus *Magnaporthe oryzae*, the causal agent of rice blast disease. Deletion of *Moatf1* gene, an ortologue of *Schizosaccharomyces pombe* ATF/CREB involved in oxidative stress response, caused retarded vegetative growth of mycelia together with reduced transcriptional level of laccases and peroxidases. As a fact, the basic leucine zipper transcription factor Moatf1 was found to be necessary for full virulence of the fungus by regulating the transcription of laccases and peroxidases to impair reactive oxygen species-mediated plant defence [81]. 

In conclusion, the reported analyses, seen as a whole, suggest a complex picture of laccase regulation phenomena whose precise molecular mechanisms through a variety of putative *cis* acting elements is only partially known. Further studies may provide additional clues to unravel the mechanism underlying laccase transcriptional regulation and to verify its general occurrence in different species.

## Figures and Tables

**Table 1.** Inducibility of Laccase Producer Basidiomycetes in Presence of Different Aromatic Compounds

	References	ABTS	Caffeic acid / caffein	Catechol	Dimethylphenol	Ferulic acid	Gallic acid	Guaiacol	Hydroquinone	Hydroxybenzoic acid	Syringic acid	Vanillin	Vanillic acid	Veratric acid	Veratryl alcohol	2,5-xylidine
*Ceriporiopsis subvermispora*	[26]															+
*Cerrena unicolor *	[27]			+	+	–			–			–	–	+		–
*Clitocybula dusenii*	[22]														+	
*Coprinus friesii*	[28]														+	
*Coriolopsis rigida*	[3]		–								+					
*Daedalea flavida*	[29] [30]						+	+							+	
*Dichomitus squalens*	[31]														+	
*Ganoderma lucidum*	[27]				–	–									+	–
*Lentinula edodes*	[32]			–						–		–				
*Lentinus strigosus*	[33]				+		+								–	
*Mycena galopus*	[34]												–	–	+	+
*Marasmius spp.*	[35] [36]					+		+								
*Nematoloma frowardii*	[22]														+	
*Phanerochaete chrysosporium*	[37]						+	+							+	
*Phlebia spp.*	[30] [38] [39]							+						+	+	+
*Pleurotus eryngii*	[40]												+	+	–	+
*Pleurotus ostreatus*	[24]	+				+		+				–	–		–	+
*Pleurotuspulmonarius*	[23]		+			+	+	+			+	+	+		+	+
*Pleurotussajor-caju*	[8]					+	+							+		+
*Pycnoporus cinnabarinus*	[41] [42]					+		–							+	+
*Pycnoporus sanguineus*	[30] [43]					+									+	+
*Rigidoporus lignosus*	[44]		+	+		+	+	+	+				+			
*Steccherium ochraceum*	[33]				+											
*Stereum hirsutum*	[28]							+							+	
*Trametes versicolor*	[27][31] [45] [5][46]			–	–	±*	+	+	+		+			–	+	+
*Volvariella volvacea*	[47]					+				+				+		+

+ laccase expression induced in presence of the corresponding compound.

– laccase expression inhibited or not induced in presence of the corresponding compound.

* Contrasting results are reported for this compound in [5] and [27].

where nothing is shown, the corresponding compound has not been tested yet for that strain.

## References

[1] Giardina P, Faraco V, Pezzella C, Piscitelli A, Vanhulle S, Sannia G (2009). Laccases: a neverending story. Cell. Mol. Life Sci.

[2] Mayer AM, Staples RC (2002). Laccase: new functions for an old enzyme. Phytochemistry.

[3] Saparrat M, Balatti PA, Martínez MJ, Jurado M (2010). Differential regulation of laccase gene expression in *Coriolopsis rigida* LPSC No. 232. Fungal Biol.

[4] Jurado M, Prieto A, Martínez-Alcal A, Martínez AT, Martínez MJ (2009). Laccase detoxi?cation of steam-exploded wheat straw for second generation bioethanol. Bioresour. Technol.

[5] Collins PJ, Dobson A (1997). Regulation of laccase gene transcription in *Trametes versicolor*. Appl. Environ. Microbiol.

[6] Galhaup C, Goller S, Peterbauer CK, Strauss J, and Haltrich D (2002). Characterization of the major laccase isoenzyme from *Trametes pubescens* and regulation of its synthesis by metal ions. Microbiology.

[7] Terrón MC, González T, Carbajo JM, Yagüe S, Arana-Cuenca A, Téllez A, Dobson AD, González AE (2004). Structural close-related aromatic compounds have different effects on laccase activity and on lcc gene expression in the ligninolytic fungus *Trametes sp*.I-62. Fungal Genet. Biol.

[8] Soden DM, Dobson AD (2001). Differential regulation of laccase gene expression in *Pleurotus sajor-caju*. Microbiology.

[9] Baldrian P, Gabriel J (2002). Copper and cadmium increase laccase activity in *Pleurotus ostreatus*. FEMS Microbiol. Lett.

[10] Manubens A, Canessa P, Folch C, Avila M, Salas L, Vicuña R (2007). Manganese affects the production of laccase in the basidiomycete *Ceriporiopsis subvermispora*. FEMS Microbiol. Lett.

[11] Faraco V, Giardina P, Sannia G (2003). Metal-responsive elements in *Pleurotus ostreatus* laccase gene promoters. Microbiology.

[12] Periasamy R, Palvannan T (2010). Optimization of laccase production by Pleurotus ostreatus IMI 395545 using the Taguchi DOE methodology. J. Basic Microbiol.

[13] Temp U, Eggert C (1999). Novel interaction between lacasse and cellobiose dehydrogenase during pigment synthesis in the white rot fungus. Appl. Environ. Microbiol.

[14] Lettera V, Piscitelli A, Leo G, Birolo L, Pezzella C, Sannia G (2010). Identification of a new member of *Pleurotus ostreatus* laccase family from mature fruiting body. Fungal Biol.

[15] Galhaup C, Haltrich D (2001). Enhanced formation of laccase activity by the white-rot fungus *Trametes pubescens* in the presence of copper. Appl. Microbiol. Biotechnol.

[16] Lorenzo M, Moldes D, Sanromán MA (2006). Effect of heavy metals on the production of several laccase isoenzymes by *Trametes versicolor* and on their ability to decolourise dyes. Chemosphere.

[17] Fan F, Zhuo R, Sun S, Wan X, Jiang M, Zhang X, Yang Y (2010). Cloning and functional analysis of a new laccase gene from *Trametes sp.* 48424 which had the high yield of laccase and strong ability for decolorizing different dyes. Bioresour. Technol.

[18] Giardina P, Palmieri G, Scaloni A, Fontanella B, Faraco V, Cennamo G, Sannia G (1999). Protein and gene structure of a blue laccase from *Pleurotus ostreatus*. Biochem. J.

[19] Alvarez JM, Canessa P, Mancilla RA, Polanco R, Santibáñez PA, Vicuña R (2009). Expression of genes encoding laccase and manganese-dependent peroxidase in the fungus *Ceriporiopsis subvermispora* is mediated by an ACE1-like copper-fist transcription factor. Fungal Genet. Biol.

[20] Palmieri G, Giardina P, Bianco C, Fontanella B, Sannia G (2000). Copper induction of laccase isoenzymes in the ligninolytic fungus *Pleurotus ostreatus*. Appl. Environ. Microbiol.

[21] Palmieri G, Bianco C, Cennamo G, Giardina P, Marino G, Monti M, Sannia G (2001). Purification characterization and functional role of a novel extracellular protease from *Pleurotus ostreatus*. Appl. Environ. Microbiol.

[22] Scheel T, Höfer M, Ludwig S, Hölker U (2000). Differential expression of manganese peroxidase and laccase in white-rot fungi in the presence of manganese or aromatic compounds. Appl. Microbiol. Biotechnol.

[23] de Souza CGM, Tychanowicz GK, de Souza D.F. Perlata RM (2004). Production of laccase isoforms by *Pleurotus pulmonarius* in response to presence of phenolic and aromatic compounds. J. Basic Microbiol.

[24] Hou H, Zhou J, Wang J, Du C, Yan B (2004). Enhancement of laccase production by *Pleurotus ostreatus* and its us. Process Biochem.

[25] Thurston CF (1994). The structure and function of fungal laccases. Microbiology.

[26] Fukushima Y, Kent Kirk T (1995). Laccase component of the *Ceriporiopsis subvermispora* lignin-degrading system. Appl. Environ Microbiol.

[27] Elisashvili V, Kachlishvili E, Khardziani T, Agathos SN (2010). Effect of aromatic compounds on the production of laccase and manganese peroxidase by white-rot basidiomycetes. J. Ind. Microbiol. Biotechnol.

[28] Heinzkill MH (1995). Isolierung under charakterisierung von Laccasen and peroxidasen aus Basidiomyceten der Ordnung Agaricales. Dissertation, Universtat Kaiserslantern.

[29] Arora DS, Sandhu DK (1985). Laccase production and wood degradation by a white rot fungus *Daedalea flavida*. Enzyme Microb. Technol.

[30] Arora DS, Gill PK (2001). Effects of various media and supplements on laccase production by some white rot fungi. Bioresour. Technol.

[31] Arora DS, Rampal P (2002). Laccase production by some *Phlebia* species. J. Basic Microbiol.

[32] Cavallazzi JRP, Oliveira MGA, Kasuya MCM (2005). Screening of inducers for laccase production by Lentinula edodes in liquid medium. Braz. J. Microbiol.

[33] Myasoedova NM, Chernykh AM, Psurtseva NV, Belova NV, Golovleva LA (2008). New effcient producers of fungal laccases. Appl. Biochem. Microbiol.

[34] Ghosh A, Frankland JC, Thurston CF, Robinson CH (2003). Enzyme production by *Mycena galopus* mycelium in arti?cial media and in Picea sitchensis F1 horizon needle litter. Mycol. Res.

[35] Farnet AM, Tagger S, Le Petit J (1999). Effects of copper and aromatic inducers on the laccases of the white-rot fungus. Marasmius quercophilus, C.R.A.S. Life Sci.

[36] Farnet AM, Criquet S, Cigna M, Gil G, Ferré E (2004). Purification of a laccase from *Marasmius quercophilus* induced with ferulic acid: reactivity towards natural and xenobiotic aromatic compounds. Enzyme Microb. Technol.

[37] Gnanamani A, Jayaprakashvel M, Arulmani M, Sadulla S (2006). Effect of inducers and culturing processes on laccase synthesis in *Phanerochaete chrysosporium* NCIM 1197 and the constitutive expression of laccase isozymes. Enzyme Microb. Technol.

[38] Arora DS, Gill PK (2000). Laccase production by some white rot fungi under different nutritional conditions. Bioresour. Technol.

[39] Rogalski J, Leonowicz A (1992). *Phlebia radiata* laccase forms induced by veratric acid and xylidine in relation to lignin peroxidases and manganese-dependent peroxidase. Acta Biotechnol.

[40] Muñoz C, Guillén F, Martínez AT, Martínez MJ (1997). Induction and characterization of laccase in the ligninolytic fungus *Pleurotus eryngii*. Curr. Microbiol.

[41] Eggert C, Temp U, Eriksson KEL (1996). The ligninolytic system of the white rot fungus *Pycnoporus cinnabarinus:* purification and characterization of the laccase. Appl. Environ. Microbiol.

[42] Meza JC, Auria R, Lomascolo A, Sigoillot J, Casalot L (2007). Role of ethanol on growth, laccase production and protease activity in *Pycnoporus cinnabarinus ss3*. Enzyme Microb. Technol.

[43] Pointing SB, Jones EBG, Vrijmoed LLP (2000). Optimization of laccase production by *Pycnoporus sanguineus* in submerged liquid culture. Mycologia.

[44] Cambria MT, Ragusa S, Calabrese V, Cambria A (2010). Enhanced laccase production in white-rot fungus *Rigidoporus lignosus* by the addition of selected phenolic and aromatic compounds. Appl. Biochem. Biotechnol.

[45] Xavier AMRB, Tavares APM, Ferreira R, Amado F (2007). *Trametes versicolor* growth and laccase induction with by-products of pulp and paper industry. Electron. J. Biotechn.

[46] Xin F, Geng A (2010). Utilization of horticultural waste for laccase production by *Trametes versicolor* under solid-state fermentation. Appl. Biochem. Biotechnol.

[47] Chen S, Ma D, Ge W. Buswell JA (2003). Induction of laccase activity in the edible straw mushroom, *Volvariella volvacea*. FEMS Microbiol. Lett.

[48] D'souza TM, Merritt CS, Reddy CA (1999). Lignin-modifying enzymes of the white rot basidiomycete *Ganoderma lucidum*. Appl. Environ. Microbiol.

[49] Vanhulle S, Enaud E, Trovaslet M, Nouaimeh N, Bols CM, Keshavarz T, Tron T, Sannia G, Corbisier AM (2007). Overlap of Laccases/Cellobiose dehydrogenase activities during the decolourisation of anthraquinonic dyes with close chemical structures by *Pycnoporus* strains. Enzyme Microb. Technol.

[50] Shuttleworth KL, Postie L, Bollag J-M (1986). Production of induced laccase by the fungus *Rhizoctonia praticola*. Can. J. Microbiol.

[51] Dekker RFH, Barbosa AM, Giese EC, Godoy SDS, Covizzi LG (2007). Influence of nutrients on enhancing laccase production by *Botryosphaeria rhodina* MAMB-05. Int. Microbiol.

[52] Vanhulle S, Radman RR, Parra RR, Cui T, Bols CM, Tron T, Sannia G, Keshavarz T (2007). Effect of mannan oligosaccharide elicitor and ferulic acid on enhancement of laccases production in liquid cultures of basidiomycetes. Enzyme Microb. Technol.

[53] Nyanhongo GS, Gomez J, Gubitz G, Zvanya R, Read JS, Steiner W (2002). Production of laccase by a newly isolated strain of *Trametes modesta*. Bioresour. Technol.

[54] Yaver DS, Xu F, Golightly EJ, Brown KM, Brown SH, Rey MW, Schneider P, Halkier T, Mondorf K, Dalboge H (1996). Purification, characterization, molecular cloning, and expression of two laccase genes from the white rot basidiomycete *Trametes villosa*. Appl. Environ. Microbiol.

[55] Xiao YZ, Hong YZ, Li JF, Hang J, Tong PG, Fang W, Zhou CZ (2006). Cloning of novel laccase isozyme genes from *Trametes sp*. AH28-2 and analyses of their differential expression. Appl. Microbiol. Biote.

[56] Chen S, Ge W, Buswell JA (2004). Molecular cloning of a new laccase from the edible straw mushroom *Volvariella volvacea:* possible involvement in fruit body development. FEMS Microbiol. Lett.

[57] Janusz G, Rogalski J, Szczodrak J (2007). Increased production of laccase by *Cerrena unicolor* in submerged liquid cultures. World J. Microbiol. Biotechnol.

[58] Mansur M, Suárez T, González AE (1998). Differential gene expression in the laccase gene family from basidiomycete I-62 (CECT 20197). Appl. Environ. Microbiol.

[59] Colao MC, Garzillo AM, Buonocore V, Schiesser A, Ruzzi M (2003). Primary structure and transcription analysis of a laccase-encoding gene from the basidiomycete *Trametes trogii*. Appl. Microbiol. Biotechnol.

[60] Mager  W.H, De Kruijff  AJ (1995). Stress-induced transcriptional activation. Microbiol Rev.

[61] Lewin B (1997). Genes VI.

[62] Thiele DJ (1992). Metal-regulated transcription in eukaryotes. Nucleic Acids Res.

[63] Strauss J, Horvath HK, Abdallah BM, Kindermann J, Mach RL, Kubicek CP (1999). The function of CreA, the carbon catabolite repressor of *Aspergillus nidulans*, is regulated at the transcriptional and post-transcriptional level. Mol. Microbiol.

[64] Litvintseva AP, Henson JM (2002). Cloning, characterization, and transcription of three laccase genes from gaeumannomyces graminis var. tritici, the take-all fungus. Appl. Environ. Microbiol.

[65] Buchman C, Scroch P, Welch J, Fogel S, Karin M (1989). The CUP2 gene product, regulator of yeast metallothionein expression, is a copper-activated DNA-binding protein. Mol. Cell. Biol.

[66] Soden DM, Dobson a DW (2003). The use of amplified flanking region-PCR in the isolation of laccase promoter sequences from the edible fungus *Pleurotus sajor-caju*. J. App. Microbiol.

[67] Marzluf G (1997). Genetic regulation of nitrogen metabolism in the fungi. Microbiol. Mol. Biol. Rev.

[68] Martin C, Pecyna M, Kellner H, Jehmlich N (2007). Purification and biochemical characterization of a laccase from the aquatic fungus *Myrioconium* sp. UHH 1-13-18-4 and molecular analysis of the laccase-encoding gene. Appl. Microbiol. Biotechnol.

[69] Tetsch L, Bend J, Hölker U (2006). Molecular and enzymatic characterisation of extra- and intracellular laccases from the acidophilic ascomycete *Hortaea acidophila*. Antonie van Leeuwenhoek.

[70] Boominathan K, Reddy CA, Arora D. K, Elander R. P, Mukerji K. G (1992). Fungal degradation of lignin: biotechnological applications. Handbook of Applied Mycology.

[71] Morgan BA, Banks GR, Toone WM, Raitt D, Kuge S, Johnston LH (1997). The Skn7 response regulator controls gene expression in the oxidative stress response of the budding yeast *Saccharomyces cerevisiae.*. EMBO J.

[72] Pezzella C, Autore F, Giardina P, Piscitelli A, Sannia G, Faraco V (2009). The *Pleurotus ostreatus* laccase multi-gene family: isolation and heterologous expression of new family members. Curr. Genet.

[73] Ogra Y, Suzuki K, Gong P, Otsuka F, Koizumi S (2001). Negative regulatory role of Sp1 in metal responsive element mediated transcriptional activation. J. Biol. Chem.

[74] Polanco R, Canessa P, Rivas A, Larrondo LF, Lobos S, Vicuña R (2006). Cloning and functional characterization of the gene encoding the transcription factor AceI in the basidiomycete *Phanerochaete chrysosporium*. Biol. Res.

[75] Canessa P, Alvarez JM, Polanco R, Bull P, Vicuña R (2008). The copper-dependent ACE1 transcription factor activates the transcription of the mco1 gene from the basidiomycete *Phanerochaete chrysosporium*. Microbiology.

[76] Jiang N, Sun N, Xiao D, Pan J, Wang Y, Zhu X (2009). A copper-responsive factor gene*CUF1* is required for copper induction of laccase in *Cryptococcus neoformans*. FEMS Microbiol. Lett.

[77] Waterman SR, Hacham M, Hu G, Zhu X, Park Y-dong, Shin S, Panepinto J, Valyi-nagy T, Beam C, Husain S, Singh N, Williamson P R (2007). Role of a CUF1/CTR4 copper regulatory axis in the virulence of *Cryptococcus neoformans*. J. Clin. Invest.

[78] Zhang S, Hacham M, Panepinto J, Hu G, Shin S, Zhu X, Williamson PR (2006). The Hsp70 member, Ssa1, acts as a DNA-binding transcriptional co-activator of laccase in *Cryptococcus neoformans*. Mol. Microbiol.

[79] Lee S, Carlson T, Christian N, Lea K, Kedzie J, Reilly J, Bonner J (2000). The yeast heat shock transcription factor changes conformation in response to superoxide and temperature. Mol. Biol. Cell.

[80] Cañero DC, Roncero M IG (2008). Functional analyses of laccase genes from *Fusarium oxysporum*. Phytopathology.

[81] Guo M, Guo W, Chen Y, Dong S, Zhang X, Zhang H, Song W, Wang W, Wang W, Lv R, Zhang Z, Wang Y, Zheng X (2010). The basic leucine zipper transcription factor Moatf1 mediates oxidative stress responses and is necessary for full virulence of the rice blast fungus *Magnaporthe oryzae*. Mol. Plant-Microbe Interact.

